# Glucocorticoid-Induced TNF Receptor Family-Related Protein Ligand is Requisite for Optimal Functioning of Regulatory CD4^+^ T Cells

**DOI:** 10.3389/fimmu.2014.00035

**Published:** 2014-02-03

**Authors:** Gongxian Liao, Michael S. O’Keeffe, Guoxing Wang, Boaz van Driel, Rene de Waal Malefyt, Hans-Christian Reinecker, Roland W. Herzog, Cox Terhorst

**Affiliations:** ^1^Division of Immunology, Beth Israel Deaconess Medical Center, Harvard Medical School, Boston, MA, USA; ^2^Biologics Discovery, Merck Research Laboratories, Palo Alto, CA, USA; ^3^Gastrointestinal Unit and Center for the Study of Inflammatory Bowel Disease, Department of Medicine, Massachusetts General Hospital, Harvard Medical School, Boston, MA, USA; ^4^Department of Pediatrics, University of Florida, Gainesville, FL, USA

**Keywords:** GITR-L, TNFSF18, Flt3L, Treg, CX3CR1

## Abstract

Glucocorticoid-induced tumor necrosis factor receptor family-related protein (TNFRSF18, CD357) is constitutively expressed on regulatory T cells (Tregs) and is inducible on effector T cells. In this report, we examine the role of glucocorticoid-induced TNF receptor family-related protein ligand (GITR-L), which is expressed by antigen presenting cells, on the development and expansion of Tregs. We found that GITR-L is dispensable for the development of naturally occurring FoxP3^+^ Treg cells in the thymus. However, the expansion of Treg in *GITR-L*^−/−^ mice is impaired after injection of the dendritic cells (DCs) inducing factor Flt3 ligand. Furthermore, DCs from the liver of *GITR-L*^−/−^ mice were less efficient in inducing proliferation of antigen-specific Treg cells *in vitro* than the same cells from *WT* littermates. Upon gene transfer of ovalbumin into hepatocytes of *GITR-L*^−/−^FoxP3(GFP) reporter mice using adeno-associated virus (AAV8-OVA) the number of antigen-specific Treg in liver and spleen is reduced. The reduced number of Tregs resulted in an increase in the number of ovalbumin specific CD8^+^ T effector cells. This is highly significant because proliferation of antigen-specific CD8^+^ cells itself is dependent on the presence of GITR-L, as shown by *in vitro* experiments and by adoptive transfers into *GITR-L*^−/−^*Rag*^−/−^ and *Rag*^−/−^ mice that had received AAV8-OVA. Surprisingly, administering αCD3 significantly reduced the numbers of FoxP3^+^ Treg cells in the liver and spleen of *GITR-L*^−/−^ but not *WT* mice. Because soluble Fc-GITR-L partially rescues αCD3 induced *in vitro* depletion of the CD103^+^ subset of FoxP3^+^CD4^+^ Treg cells, we conclude that expression of GITR-L by antigen presenting cells is requisite for optimal Treg-mediated regulation of immune responses including those in response during gene transfer.

## Introduction

CD4^+^CD25^+^FoxP3^+^ regulatory T cells (Treg), which develop in the thymus or can be induced in peripheral organs, control many aspects of the immune response (1–4). Tregs constitutively express glucocorticoid-induced tumor necrosis factor receptor family-related protein (GITR, TNFRSF18, CD357), which is inducible on effector T cells (Teffs) (2, 5–8). Using Fc-GITR-L, a soluble form of the natural ligand of GITR, we found recently that glucocorticoid-induced TNF receptor family-related protein ligand (GITR-L) preferentially induces the *in vivo* and *in vitro* expansion of functionally competent Tregs (9). Furthermore, a significantly higher proportion of FoxP3^+^ Tregs is also found in GITR-L transgenic mouse strains, in which the expression of GITR-L is under control of the CD19- and MHC-II-promoter respectively (10, 11). GITR-L is not expressed by T cells (8), but is found on plasmacytoid dendritic cells (pDCs), Langerhans cells, macrophage subpopulations, and endothelial cells (12–15). Here we use *GITR-L*^−/−^ mice to examine the role of GITR-L in the induction of Tregs and Treg-mediated suppression in response to hepatic gene transfer with the adeno-associated viral vector AAV8.

Tolerance induction to specific foreign protein by hepatic gene transfer may be established in two steps. First, antigen-specific Tregs are *de novo* induced in the hepatic microenvironment. Second, antigen-specific Tregs are expanded systemically. Indeed, we previously found that transgene product-specific Treg actively suppresses antibody and T cell responses thereby ensuring long-term gene expression (16). Recently, studies in hemophilic mouse models have shown that AAV-mediated hepatic gene transfer can not only prevent but also reverse pathogenic antibody responses and desensitize from severe allergic reactions to the therapeutic coagulation factor IX protein (17–20). We have recently shown that the immune suppressive cytokine TGF-β is required for Treg induction in hepatic AAV gene transfer and thus necessary for suppression of antibody and CD8^+^ T cell responses against the transgene product (21). TGF-β, a cytokine highly expressed in mucosal tissues and sites of inflammation, plays a role in conversion of conventional peripheral CD4^+^ T cells into Treg, and TGF-β up-regulates expression of CD103 (Integrin α_E_β_7_) (22), which is the primary ligand of E-cadherin, an epithelial adhesion molecule. Expression of CD103 marks a subset of peripheral inducible Tregs (about 20–30% of the CD4^+^FoxP3^+^ Tregs in the spleen), which inhibit graft-versus-host disease more potently than the CD4^+^CD25^+^ Tregs (23, 24).

In this study, we provide evidence in support of the concept that the interactions between GITR and GITR-L are requisite for optimal functioning of Tregs. To this end, we analyze *GITR-L*^−/−^FoxP3(GFP) and *GITR-L*^−/−^CX3CR1(GFP) mice after gene transfer of ovalbumin into hepatocytes with adeno-associated virus (AAV8-OVA). Coordinate expansion of Treg and dendritic cells (DCs) was assessed after injection of Flt3 ligand in *GITR-L*^−/−^ mice. The interactions between antigen presenting cells and Tregs are also evaluated after administering αCD3 in *GITR-L*^−/−^ mice or by co-activation with αCD3 and soluble Fc-GITR-L.

## Materials and Methods

### Mice

B6, OT-II Tg, and CX3CR1(GFP) reporter mice were purchased from the Jackson Laboratory (Bar Harbor, ME, USA). OT-I × *Rag^-/-^* mice were purchased from Taconic Labs (Germantown, NY, USA). *GITR-L^-/-^* and FoxP3-IRES-EGFP-SV40 knock-in [FoxP3(GFP)] B6 mice were described previously (8, 25). *GITR-L^-/-^* mice were crossed with FoxP3(GFP) and CX3CR1(GFP) mice to generate *GITR-L^-/-^*FoxP3(GFP) and *GITR-L^-/-^*CX3CR1(GFP) B6 mice. All animals were housed in the Center for Life Science animal facility of BIDMC. The Guide for the Care and Use of Laboratory Animals was followed in the conduct of the animal studies of the Institutional Animal Care and Use Committee at BIDMC. Veterinary care was given to any animals requiring medical attention.

### Antibodies

Anti-CD11b-PacBlu, αCD11b-FITC, αCD4-PE, αCD4-APC, αCD11c-APC, αCD11c-PE, αTCRvα2-PE, and αCD3ε(145-2C11) were purchased from BioLegend (San Diego, CA, USA). Anti-Ly6C-PerCP and αFoxP3-APC were products of eBioscience (San Jose, CA, USA). Anti-Ly6G-PE, αNK1.1-PE, αCD8α-PacBlu, αCD25-PE, and αCD103-Alexa Fluor 647 were products from BD Biosciences (San Jose, CA, USA). Flt3L-Fc fusion protein was purchased from BioXCell (West Lebanon, NH, USA). Anti-IL-2 was purchased from R&D Systems (Minneapolis, MN, USA). Fc-GITR-L fusion protein was produced as described previously (9).

### AAV8-OVA mediated expression of foreign protein in hepatocytes

AAV8-OVA vector (containing an ovalbumin expression cassette driven by AAV-EF1α) was packaged into serotype 8 capsid as described previously (16). Vector was injected *i.v*. into FoxP3(GFP) and *GITR-L^-/-^*FoxP3(GFP) mice at a dose of 10^10^ vector genome/mouse. Five weeks later, leukocytes from liver, spleen, and thymus were stained with TCRvα2.

Also, Ly6G^-^NK1.1^-^GFP^+^ cells FACS sorted from the liver of CX3CR1(GFP) mice 7 days after AAV8-OVA injection were incubated with OT-II CD4^+^ or CFSE-labeled OT-I CD8^+^ T cells for 3 days. OT-II CD4^+^ T cell cultures were stained with TCRvα2 and FoxP3. OT-I CD8^+^ T cell culture was stained with TCRvα2 and proliferating CD8^+^ cells were evaluated by CFSE dilution.

### Induction of dendritic cells and Treg with Flt3L

Flt3L-Fc fusion protein (10 ng/mouse/injection) was *i.p*. injected into FoxP3(GFP) and *GITR-L^-/-^*FoxP3(GFP) mice for nine consecutive days as described previously (26). Leukocytes from the spleen and liver were analyzed at day 10.

### Cellularity in mice after αCD3-mediated activation of T cells by *in vivo*

Anti-CD3ε was *i.p*. injected into CX3CR1(GFP) and *GITR-L*^−/−^CX3CR1(GFP) mice (20 μg/mouse, one injection). After 72 h, leukocytes of the spleen and liver were stained with CD4 and FoxP3. CX3CR1^+^ cells were evaluated by expression of the reporter gene GFP.

### *In vitro* activation of CD4^+^ T cells

CD4^+^ T cells from the spleen of FoxP3(GFP) mice were negatively selected using a CD4^+^ T cells isolation kit (Miltenyi, Auburn, CA, USA) and were activated with αCD3-coupled microbeads in a round bottom 96-well plate in the presence or absence of Fc-GITR-L (1 μg/ml) for 2 days as described previously (9). Cells were stained with CD4 and CD103. Expression of FoxP3 was judged by the reporter protein EGFP. Cell numbers were counted with a Countess Automated Cell Counter (Invitrogen, Grand Island, NY, USA).

### Isolation of liver leukocytes

Liver leukocytes were isolated as described previously (27). Briefly, liver was mashed and filtered through a 70 μM cell strainer. Hepatocytes and cell debris were removed by spinning at 300 rpm for 10 min. Supernatant was centrifuged at 1500 rpm for 10 min to collect cells. Leukocytes were isolated from the interface of a 40 and 70% Percoll gradient.

Statistical analysis used Prism 4.0c software (GraphPad, San Diego, CA, USA). Statistical comparisons were performed using the two-tailed Student’s *t*-test. Values of *P* < 0.05 were considered to be statistically significant.

## Results

### Flt3L-induced expansion of Treg was impaired in GITR-L deficient mice due to a partially reduced number of dendritic cell subpopulations

**Figure 1:**
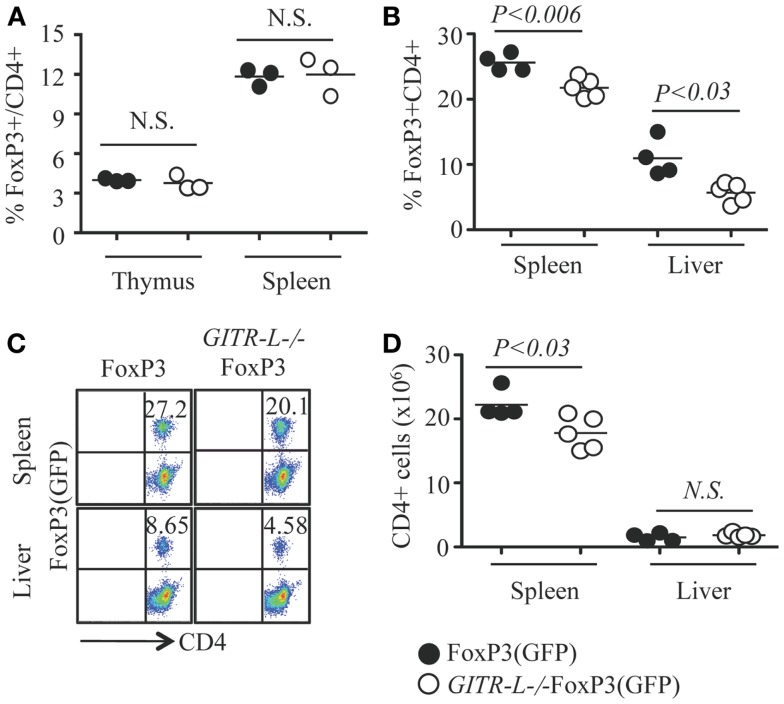
**Flt3L-induced expansion of Treg**. Flt3L-Fc fusion protein was injected into FoxP3(GFP) and *GITR-L*^−/−^FoxP3(GFP) mice (10 ng/mouse/injection, nine injections). CD4^+^CD8^−^ T cells from the thymus, spleen, and/or liver were analyzed by FACS for FoxP3 expression based on the expression of reporter protein EGFP. **(A)** Percentages of CD4^+^FoxP3^+^ Treg in the thymus and spleen of FoxP3(GFP) and *GITR-L*^−/−^FoxP3(GFP) mice without stimulation. Percentages **(B)** and representative staining **(C)** of Treg in the spleen and liver after administering Flt3L. **(D)** Number of CD4^+^ T cells in the spleen and liver of FoxP3(GFP) and *GITR-L*^−/−^FoxP3(GFP) mice after administering Flt3L. Filled circle represents FoxP3(GFP) mouse. Open circle represents *GITR-L*^−/−^FoxP3(GFP) mouse. Each circle represents one mouse.

We previously found that after administering a Fc-GITR-L fusion protein to *WT* mice the number of Treg cells increased, which was confirmed by studies with GITR-L transgenic mice (9–11, 28). Surprisingly, we found that GITR-L was dispensable for the development of naturally occurring Treg, as the number of FoxP3^+^ Treg cells was normal in the thymus and spleen of *GITR-L*^−/−^FoxP3(GFP) mice under resting conditions (Figure 1A; Figure S1 in Supplementary Material).

To further investigate the role of GITR-L in controlling Treg development, we assessed the consequences of injecting Fms-related tyrosine kinase 3 ligand (Flt3L) into FoxP3(GFP) and *GITR-L*^−/−^FoxP3(GFP) mice for nine consecutive days. Not only is Flt3L a potent inducer of DC and macrophage proliferation (26, 29), several phagocyte subpopulations express GITR-L (12, 30). After the injection of Fc-Flt3L fusion protein, both the numbers and the frequency of FoxP3^+^ Treg were significantly increased in the spleen and liver. This Fc-Flt3L-induced expansion was, however, significantly reduced in *GITR-L*^−/−^FoxP3(GFP) mice (Figures 1B,C). The total number of CD4^+^ T cells in the spleen was also lower in *GITR-L*^−/−^FoxP3(GFP) mice than the *WT* counterparts (Figure 1D). Thus, GITR-L plays a significant role in the expansion of Treg in the peripheral tissues.

**Figure 2:**
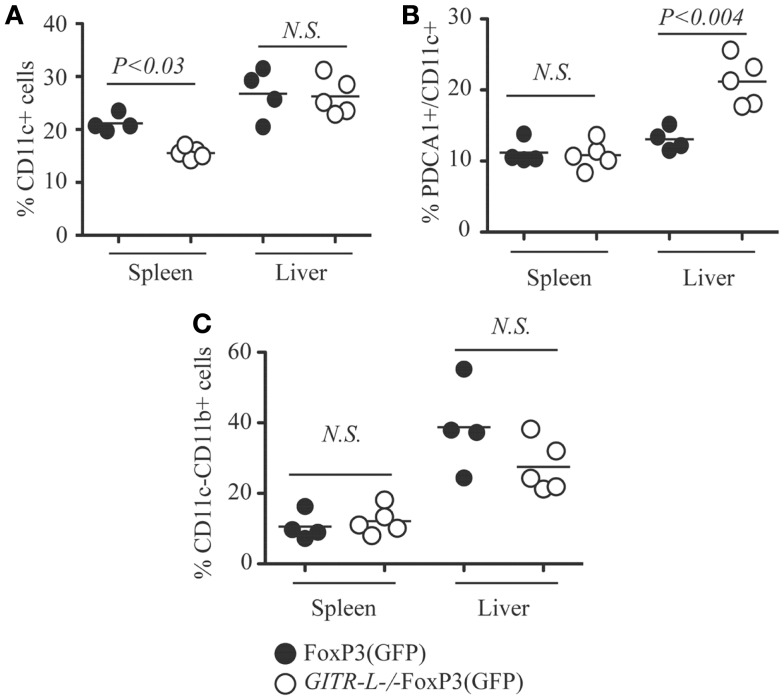
**CD11c^+^ DCs after Flt3L induction**. Flt3L-Fc fusion protein was injected into FoxP3(GFP) and *GITR-L*^−/−^FoxP3(GFP) mice as described in Figure 1. Different subsets of myeloid cells in the spleen and liver were analyzed. **(A)** Percentages of CD11c^+^ cells in the spleen and liver. **(B)** Percentages of CD11c^+^PDCA1^+^ cells in the spleen and liver. **(C)** Percentages of CD11c^−^CD11b^+^ cells. Filled circle represents FoxP3(GFP) mouse. Open circle represents *GITR-L*^−/−^FoxP3(GFP) mouse. Each circle represents one mouse.

We next evaluated whether the impaired Flt3L-induced expansion of Treg cells in *GITR-L*^−/−^FoxP3(GFP) mice correlated with reduced numbers of DCs and macrophages (MØ) (31, 32). As shown in Figure 2A and Figure S2A in Supplementary Material, the percentage of CD11c^+^CD11b^+^ and CD11c^+^CD11b^−^ DCs was reduced in the spleen of *GITR-L*^−/−^FoxP3(GFP) mice as compared to FoxP3(GFP) mice. Although the number of conventional CD11c^+^ DCs in the liver was normal (Figure 2A), the percentage of pDCs in *GITR-L*^−/−^FoxP3(GFP) mice was higher than that of their *WT* counterparts (Figure 2B; Figure S2B in Supplementary Material and Data not shown). The frequency of CD11c^−^CD11b^+^ MØ was comparable between these two mice (Figure 2C). Taken together, these data indicate that after Flt3L induction, GITR-L affects the expansion and differentiation of subpopulations of DCs, which in turn leads to expansion of Tregs.

### GITR-L^−/−^ CX3CR1^+^ DCs isolated from the liver are less efficient than WT CX3CR1^+^ DCs in the *in vitro* induction of OVA-specific Treg and CD8^+^ T cells

**Figure 3:**
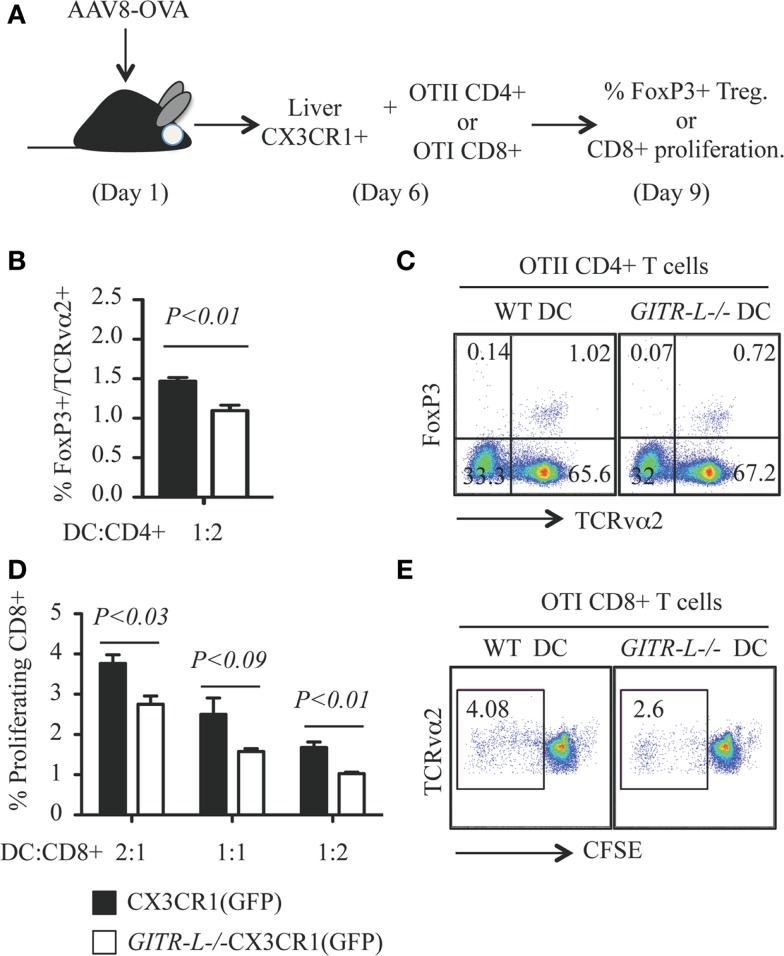
***In vitro* induction of OVA-specific Treg and CD8^+^ T cells with hepatic CX3CR1^+^ DCs**. **(A)** Schematic for *in vitro* priming of CD8^+^ OT-I and CD4^+^ OT-II T cells. Briefly, AAV8-OVA(10^10^ vector genome/mouse) was *i.v*. injected into CX3CR1(GFP) and *GITR-L*^−/−^CX3CR1(GFP) mice. After 7 days, Ly6G-NK1.1-CX3CR1(GFP)^+^ cells were purified from the liver and incubated with CD8^+^ OT-I T and CD4^+^ OT-II T cells at different ratios for 3 days. Divisions of CD8^+^ OT-I T cells were evaluated by CFSE dilution. **(B)** Percentages of *in vitro* induced of TCRvα2^+^FoxP3^+^ Treg. **(C)** Representative stainings of **(B)**. **(D)** Ratios of proliferating TCRvα2^+^CD8^+^ OT-I T cells. **(E)** Representative staining of **(D)**. Results represent one of three independent experiments. Error bars indicate mean ± SEM of triplicates.

To directly test whether the absence of GITR-L in DC subpopulations affects proliferation of antigen-specific GITR^+^ Treg and CD8^+^ cells, we immunized *GITR-L*^−/−^CX3CR1(GFP) and *WT* CX3CR1(GFP) mice by gene transfer with AAV8-OVA (Figure 3A). One week after injection of AAV8-OVA, liver CX3CR1(GFP)^+^ cells purified by FACS were incubated with OVA-specific OT-II CD4^+^ T cells or OT-I CD8^+^ cells for 3 days. *GITR-L*^−/−^ CX3CR1^+^ cells were less efficient in inducing Treg as compared to the same cells isolated from *WT* mice (Figures 3B,C). Since activated CD8^+^ cells carry GITR on their surface, we also evaluated whether *in vitro* proliferation of CD8^+^ T cells would be affected by the absence of GITR-L from the surface of these DCs. Indeed, the proliferation of CD8^+^ OT-I cells was reduced when cocultured with liver CX3CR1^+^ cells from AAV8-OVA-primed *GITR-L*^−/−^CX3CR1(GFP) mice compared to OT-I cells cultured with *WT* CX3CR1^+^ DCs (Figures 3D,E).

We conclude that GITR-L on the surface of antigen presenting cells can drive proliferation of both FoxP3^+^CD4^+^ Treg cells and activated CD8^+^ T cells in an antigen-specific manner.

### After AAV8-OVA gene transfer, the number of antigen-specific Treg in GITR-L^−/−^FoxP3 mice is reduced, which results in an increased number of OVA-specific CD8^+^ T cells

**Figure 4:**
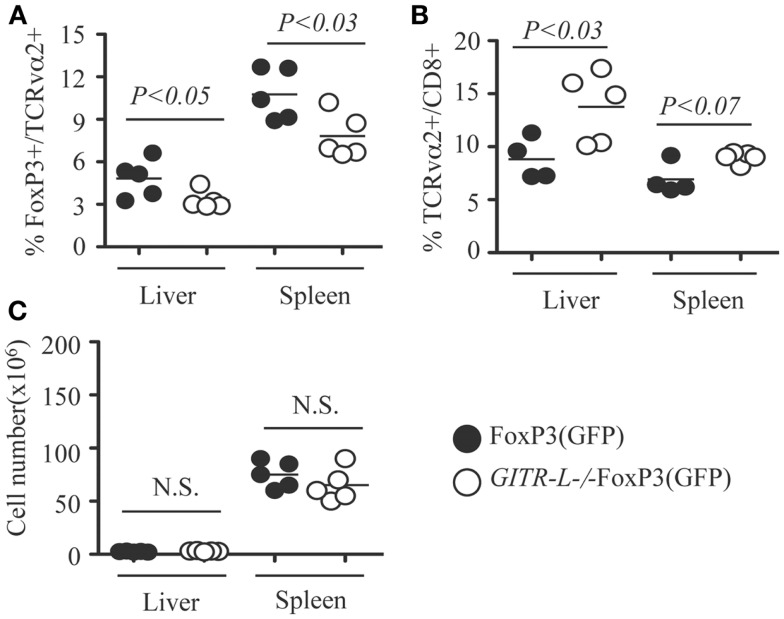
***In vivo* induction of OVA-specific Treg and CD8^+^ T cells in *GITR-L*^−/−^FoxP3(GFP) mice**. AAV8-OVA (10^10^ vector genome/mouse) was *i.v*. injected into FoxP3(GFP) and *GITR-L*^−/−^FoxP3(GFP) mice. Five weeks later, leukocytes from the liver and spleen were stained with TCRvα2. **(A)** Percentages of FoxP3^+^/TCRvα2^+^ cells. **(B)** Percentages of TCRvα2^+^/CD8^+^ cells. **(C)** Number of leukocytes. Filled circle represents FoxP3(GFP) mouse. Filled circle represents FoxP3(GFP) mouse. Open circle represents *GITR-L*^−/−^FoxP3(GFP) mouse. Each circle represents one mouse.

Because targeted expression of exogenous protein in hepatocytes by AAV8-mediated gene transfer induces a Treg-mediated tolerance (16), we assessed whether this process involves GITR-L. To assess this, we injected an AAV8-OVA vector into in FoxP3(GFP) and *GITR-L*^−/−^FoxP3(GFP) mice and determined the number of OVA-specific Treg and CD8^+^ T cells. Consistent with the results when administering Flt3L, there was a reduced percentage of OVA-specific FoxP3^+^TCRvα2^+^ T cells in the spleen and liver of *GITR-L*^−/−^FoxP3(GFP) mice as compared to that of *WT* mice 5 weeks after vector administration (Figure 4A). Conversely, AAV-mediated OVA expression in the hepatocytes induced an increased percentage of OVA-specific CD8^+^TCRvα2^+^ T cells in the spleen and liver of *GITR-L*^−/−^FoxP3(GFP) mice (Figure 4B). By contrast, the total cell numbers were comparable between these two mouse strains (Figure 4C). The data suggest that GITR-L deficiency may impair the induction of antigen-specific Tregs (16–18, 21, 33), which may at least partially compromise their immunosuppressive capability.

**Figure 5:**
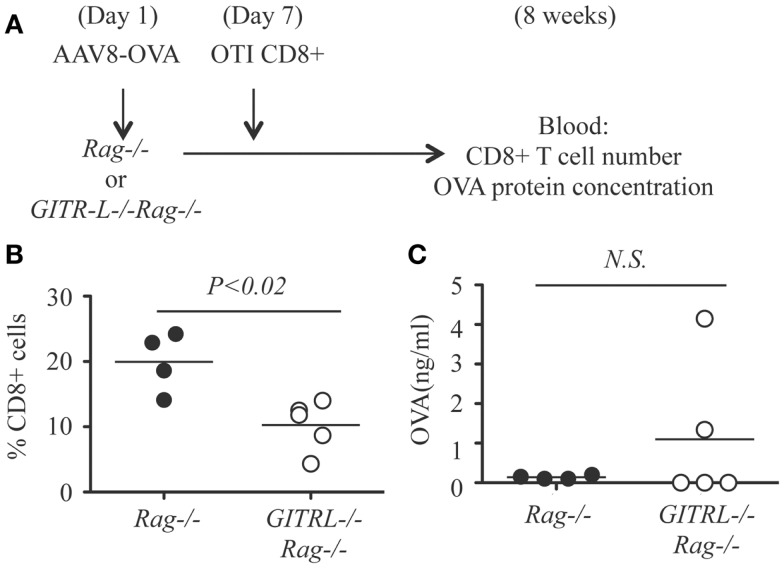
***In vivo* induction of OVA-specific CD8^+^ T cells in *GITR-L*^−/−^*Rag*^−/−^ mice**. **(A)** Schematic for *in vivo* expansion of CD8^+^ OT-I T cells. Briefly, AAV8-OVA (10^10^ vector genome/mouse) vector was *i.v*. injected to *Rag*^−/−^ or *GITR-L*^−/−^*Rag*^−/−^ mice. Seven days later, CD8^+^ OT-I T cells (10^6^ cells/mouse) were *i.p*. injected to mice with AAV8-OVA gene transfer. Mice were euthanized after 8 weeks. **(B)** Percentages of CD8^+^ T cells in the blood were evaluated by FACS. **(C)** Concentrations of chicken ovabulmin protein in the plasma were measured by ELISA. Filled circle represents *Rag*^−/−^ mouse. Open circle represents *GITR-L*^−/−^*Rag*^−/−^ mouse. Each circle represents one mouse.

As the *in vitro* data suggest that GITR-L expression on DCs causes the expansion of CD8^+^ cells, this *in vivo* result might underestimate the consequences of the reduced number of the Tregs in the *GITR-L*^−/−^ mice. To test whether GITR-L is implicated in the *in vivo* expansion of antigen-specific CD8^+^ cells, we used a system in which the Treg-mediated suppression is absent. To this end, we injected AAV8-OVA into *Rag*^−/−^ and *GITR-L*^−/−^*Rag*^−/−^ mice followed by the adoptive transfer of OT-I CD8^+^ T cells after 1 week (Figure 5A). Eight weeks after transfer of OT-I CD8^+^ T cells, the number of CD8^+^ T cells in the blood of the *GITR-L*^−/−^*Rag*^−/−^ recipients was significantly lower than that of the *Rag*^−/−^ recipients (Figure 5B). This was not due to an inadequate amount of OVA antigen production in the *GITR-L*^−/−^*Rag*^−/−^ recipients (Figure 5C). Taken together, the data indicate that GITR-L is required for optimal induction and/or expansion of antigen-specific Treg in the context of hepatic AAV8 gene transfer.

### Depletion of CX3CR1^+^ (GFP) cells by αCD3 in GITR-L^−/−^ mice correlates with a reduced number of FoxP3^+^ Treg cells

**Figure 6:**
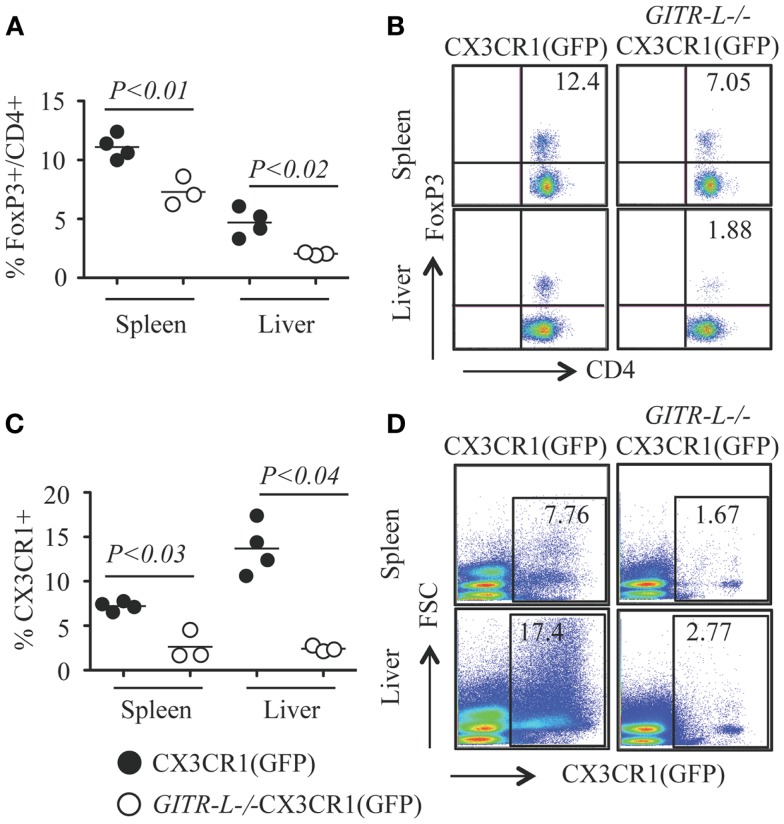
**Numbers of Treg and CX3CR1^+^ DCs after administering αCD3**. Anti-CD3ε (20 μg/mouse, one injection) was *i.p*. injected into CX3CR1(GFP) and *GITR-L*^−/−^CX3CR1(GFP) mice. Mice were euthanized at day 3. Splenocytes and liver leukocytes were stained for the expression of CD4 and FoxP3 by FACS. CX3CR1^+^ cells were assessed by the expression of reporter protein EGFP. Percentages **(A)** and representative staining **(B)** of FoxP3^+^CD4^+^ Treg cells in the spleen and liver. Percentages **(C)** and representative staining **(D)** of CX3CR1^+^ phagocytes in splenocytes and liver leukocytes. Filled circle represents CX3CR1(GFP) mouse. Open circle represents *GITR-L*^−/−^CX3CR1(GFP) mouse. Each circle represents one mouse.

*In vitro* expansion of FoxP3^+^ Treg cells can be achieved by stimulation with a combination of αCD3 and soluble GITR-L (Fc-GITR-L) (9). We then assessed whether injection of αCD3 into *WT* and *GITR-L*^−/−^ mice would affect the Treg population. As shown in Figures 6A,B, αCD3 induced a significant reduction of the percentage of FoxP3^+^ Treg in the spleen and liver of *GITR-L*^−/−^CX3CR1(GFP) mice, but not in *WT* CX3CR1(GFP) mice. In support of our observations in this paper, the reduced number of Tregs coincided with a reduction of CX3CR1^+^ DCs in the spleen and liver of *GITR-L*^−/−^CX3CR1(GFP) mice (Figures 6C,D). In contrast, the numbers of CX3CR1^+^ cells in the spleen and liver were comparable in the two mouse strains under homeostasis (Figure S3 in Supplementary Material).

**Figure 7:**
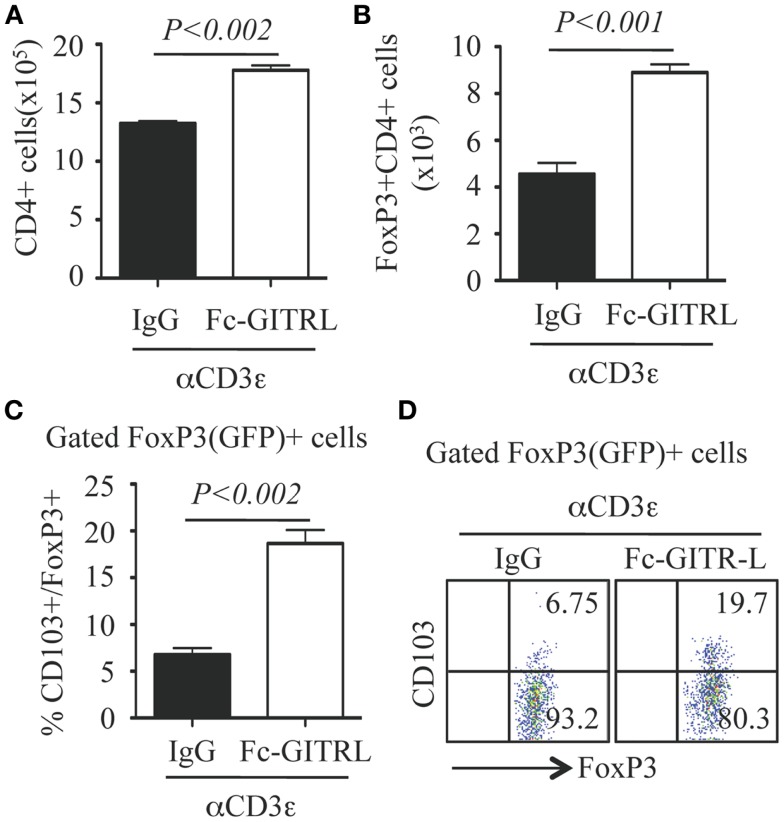
**Expansion of FoxP3^+^ and CD103^+^ Tregs by Fc-GITR-L *in vitro***. CD4^+^ T cells (10^5^ cells/well) from the spleen of FoxP3(GFP) mice were stimulated with αCD3-beads in the presence of Fc-GITR-L or control IgG for 2 days and then stained with CD4 and CD103. FoxP3 expression was judged according to the expression of reporter protein EGFP. **(A)** Number of total CD4^+^ cells. **(B)** Number of FoxP3^+^CD4^+^ Tregs. **(C)** Percentages of CD103^+^ cells among gated FoxP3^+^ Tregs. **(D)** Representative staining of **(C)**. Results were representative of at least three independent experiments. Error bars indicate mean ± SEM of triplicates.

To further investigate the role of GITR-L in the expansion of FoxP3^+^ Treg, CD4^+^ T cells were purified from the spleen of FoxP3(GFP) mice and stimulated *in vitro* with αCD3 with either Fc-GITR-L or IgG. Forty-eight hours after exposure to αCD3, the number of total CD4^+^ and FoxP3^+^CD4^+^ Treg was significantly higher in the presence of Fc-GITR-L than that of IgG (Figures 7A,B). Interestingly, a subset of CD103^+^ Treg cells, which is induced in epithelium and in sites of inflammation (23, 34) and comprises approximately 20% of all FoxP3^+^ Treg cells in the spleen, was also expanded by Fc-GITR-L (Figures 7C,D).

We conclude that while the induction or expansion of Treg is impaired in the absence of GITR-L, Fc-GITR-L provides a positive signal to GITR^+^ Treg.

## Discussion

The receptor-ligand pair GITR/GITR-L (TNFRSF18/TNFSF18) appears to be involved in the development of a variety of inflammation-related diseases in murine models (6, 8, 12, 35, 36). It was originally thought that the suppressor function of Treg cells, which constitutively express GITR, would be abrogated by anti-GITR thus breaking immune self-tolerance (2). More recent additional evidence shows that GITR engagement by its natural ligand GITR-L causes an extensive expansion of functionally competent Tregs (9–11), although the relative role of GITR on Treg and Teff cells remains only partly understood. In this study we find that in the absence of GITR-L the expansion of FoxP3^+^ Treg cells is impaired in an antigen-specific manner, which can be mimicked by *in vivo* and *in vitro* activation of CD4^+^ Treg cells with αCD3. Our results are consistent with the findings of the Chatila group that expansion and contraction of Teff and Treg dynamically control primary immune responses to foreign antigen (25).

Glucocorticoid-induced TNF receptor family-related protein ligand impacts immune regulation in gene replacement therapy at least at three levels. First, the induction/expansion of antigen-specific Treg cells in the liver after AAV-mediated gene therapy is impaired directly by the absence of GITR-L. Second, the expansion of antigen-specific CD8^+^ T cells is reduced by GITR-L deficiency. However, impaired expansion of Treg cells can on the other hand up-regulate CD8^+^ T cell expansion indirectly. Third, GITR-L deficiency affects the infiltration of monocyte-derived MØ to the sites where exogenous protein is expressed and/or the sites of inflammation (30), which changes the local function of different immune cells. These GITR-L-expressing, monocyte-derived MØ may provide a microenvironment for the expression of CD103 in Treg cells, an integrin that facilitates the retention of Treg cells in the sites of inflammation or infection.

Surprisingly, we found that administering αCD3 causes the depletion of CX3CR1^+^ DCs in the spleen and liver of *GITR-L*^−/−^ mice, which correlates with a reduced number of FoxP3^+^ Tregs. It is reported that IL10-secreting GITR^+^ Tr1 cells may suppress immune responses by granzyme B-mediated killing of myeloid APCs (37, 38). Granzyme B is also important for the ability of Treg, NK cells, and CD8^+^ T cells to kill their targets (39). It is possible that Tr1, Treg, and CD8^+^ T cells play a role in the depletion of CX3CR1^+^ DCs in *GITR-L*^−/−^ mice. In the presence of GITR-L, an increased expansion of Treg may inhibit this self-destructive cytotoxicity. Depletion of CX3CR1^+^ DCs, which includes the GITR-L-expressing pDCs and MØ (12, 30), may feedback to cause the reduction of Treg number during immune responses.

Ly6C^hi^ monocytes give rise to CX3CR1^+^ DCs under both steady state and inflammation. Under resting conditions, CX3CR1^+^ DCs in the intestine is reported to induce a immunosuppressive CD8^+^ T cells (40). CX3CR1^+^ DCs isolated from the liver are able to induce Treg *in vitro*. However, during inflammation CX3CR1^+^ DCs give rise to proinflammatory effector cells (41). The mechanism how this Ly6C^hi^ monocyte-derived DC subpopulation is educated to be either protagonist or antagonist is still not well understood. Anti-CD3-mediated depletion of CX3CR1^+^ DCs in the liver may provide an important tool for the study of migration, colonization, and education of this special DC subset (30).

In conclusion, our data show that GITR and GITR-L have important implications for gene therapy. Optimal induction of an immune regulatory response, which is crucial for tolerance to the transgene product and for immune modulatory gene therapy, requires co-stimulation by GITR-L, which enhances Treg induction and function. Expression of GITR-L on hepatic APCs may in part explain the tolerogenic/Treg inducing capacity of hepatic gene transfer.

## Author Contributions

Gongxian Liao performed all the experiments; Michael S. O’Keeffe helped in processing the samples and editing the manuscript; Guoxing Wang and Boaz van Driel helped in processing the samples and discussing the results. Rene de Waal Malefyt generated GITR-L deficient mice; Hans-Christian Reinecker brought deeper insight into αCD3-inducing murine model. Roland W. Herzog helped in discussing and writing the manuscript; Cox Terhorst is the major organizer of this work and designed the experiments with Gongxian Liao.

## Conflict of Interest Statement

The authors declare that the research was conducted in the absence of any commercial or financial relationships that could be construed as a potential conflict of interest.

## Supplementary Material

The Supplementary Material for this article can be found online at http://www.frontiersin.org/Journal/10.3389/fimmu.2014.00035/abstract

Figure S1 **Representative staining of Figure 1A**.

Figure S2 **(A,B) Representative staining of Figures 2A,B**.

Figure S3 **CX3CR1(GFP)+ phagocytes in spleen and liver leukocytes of CX3CR1(GFP) and *GITR-L*^−/−^CX3CR1(GFP) mice under resting condition**.
