# Supplementary figures and images for: Glucocorticoid-Induced TNF Receptor Family-Related Protein Ligand is Requisite for Optimal Functioning of Regulatory CD4+ T Cells
Source: Front Immunol. 2014 Feb 3;5:35. doi: 10.3389/fimmu.2014.00035 (PMC3909995; doi:10.3389/fimmu.2014.00035)

Supplement Fig. S1

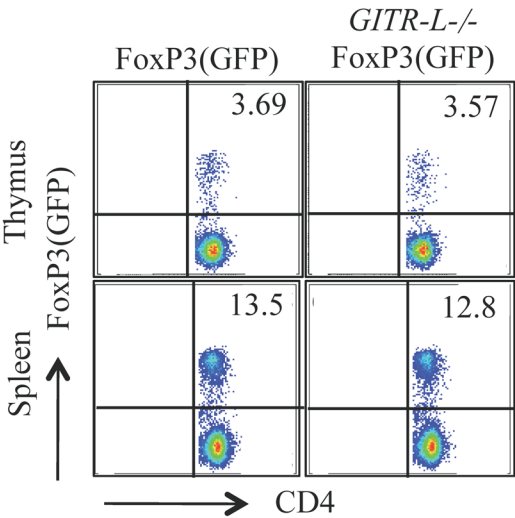

Representative staining of **Figure 1A**.

Supplement: Figure S1 — Representative staining of Figure 1A. [file 75202_Liao_Presentation1.PDF]

Supplement Fig. S2

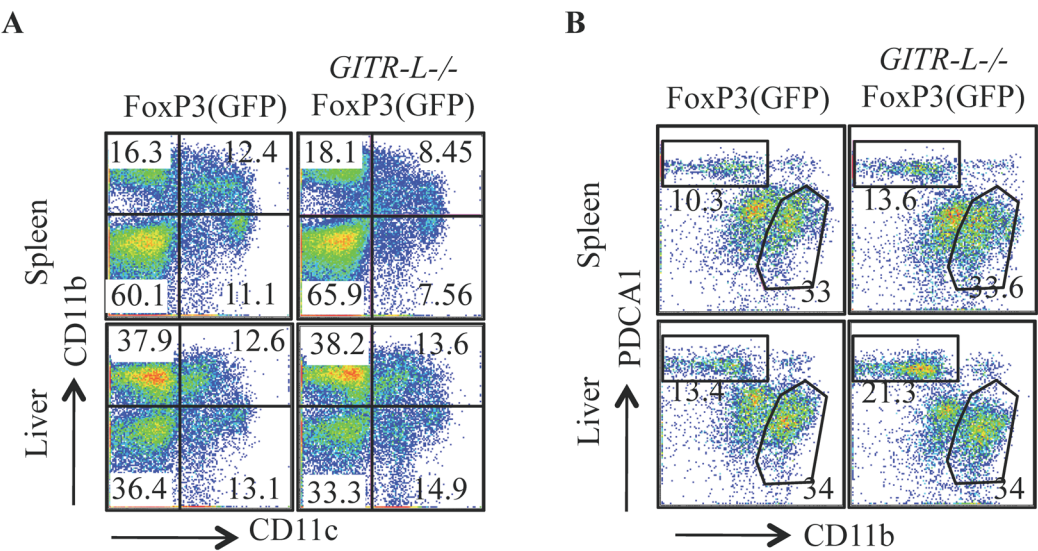

S2A & S2B, Representative staining of **Figure 2A & 2B**.

Supplement: Figure S2 — (A,B) Representative staining of Figures 2A,B. [file 75202_Liao_Presentation2.PDF]
