# Supplementary material for: Glucocorticoid-Induced TNF Receptor Family-Related Protein Ligand is Requisite for Optimal Functioning of Regulatory CD4+ T Cells
Source: Front Immunol. 2014 Feb 3;5:35. doi: 10.3389/fimmu.2014.00035 (PMC3909995; doi:10.3389/fimmu.2014.00035)
Supplement: Figure S3 — CX3CR1(GFP)+ phagocytes in spleen and liver leukocytes of CX3CR1(GFP) and GITR-L−/−CX3CR1(GFP) mice under resting condition. [file 75202_Liao_Presentation3.PDF]

Supplement Fig. S3

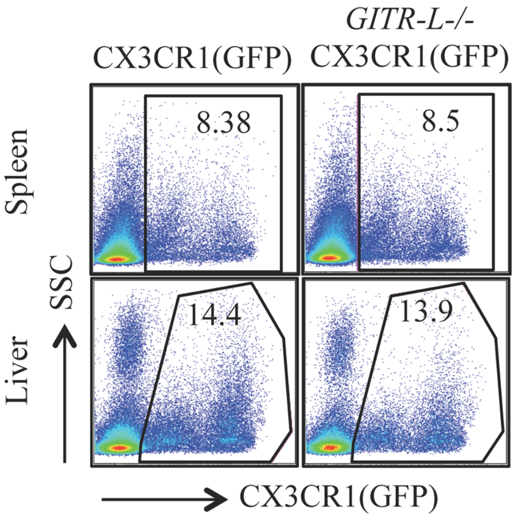

S3, CX3CR1(GFP)+ phagocytes in spleen and liver leukocytes of CX3CR1(GFP) and *GITR-L-/-*CX3CR1(GFP) mice under resting condition.
